# Editorial

**Published:** 1901-07-15

**Authors:** 


### EDITORIAL.

The editor of *Items of Interest* in the June issue of that journal raises the question as to a choice of terms descriptive of the lateral contact surfaces of the teeth, citing as an illustration of the perplexing variety in customary usage the fact that in a symposium which recently appeared in his journal there was no substantial agreement among several eminent contributors to the discussion. Some used the term "approximal," some "approximate," others "proximal," and still others "proximate." If the editor had taken the trouble to refer to the journal files of a few years ago he would have found the prefixes "anterior" and "posterior" used in connection with these same terms. And these prefixes, while objectionable because of their tautological redundancy, were nevertheless expressive and specific. It is not likely that we shall ever come to any unanimous agreement in an effort to use these terms, for the reason that they express such slight variations of meaning, as technically applied, that there is no great inducement to make a discrimination in writing or speaking necessary. In themselves, and for technical purposes, they are superfluous terms, as they add little or nothing in locating that particular part of the tooth to which they are applied. In fact, they are a great incumbrance to clear speaking and writing. and our nomenclature would be much better off without them. The terms "distal" or "mesial" are simple, and sufficient to locate the anterior or posterior, proximal, proximate, approximal or approximate surfaces of the teeth, and no one should ever confuse these terms with those used to describe the other surfaces of the teeth. The term inter-proximal space seems to have some justification for its use, but the term inter-dental space is equally significant and more appropriate. Instead, therefore, of adopting the suggestion of the editor of *Items of Interest*, especially during this hot weather, we suggest that the terms "mesial" and "distal" supersede all other terms that may have heretofore been used to describe the lateral contact surfaces of the teeth as normally placed in the dental arch.

---

THE editor of the *Dental Digest* in commenting on the Tri-State convention, make the criticism that there were too many papers read, and that time was not given to their discussion. This was true, there were, however, but eight papers, and two papers had to be read at each session. The papers were upon live topics and well written and no doubt many would have been pleased to comment upon them. One paper before such an audience would have been ample. We are wondering what kind of a time they had at the joint Maryland and Washington meeting recently held, where twenty-one papers are reported to have been read.

---

IT is encouraging to note the extended commendatory notices made upon the death of Dr. H. J. McKellops. The dental societies everywhere and all dental journals have expressed the sentiment that seems to prevail everywhere that he was a worthy exponent of our beloved profession. He is one of the men who will be missed but not forgotten, and we honor ourselves in giving this public expression to our estimate of his professional and personal career.

